# Fabrication of Myogenic Engineered Tissue Constructs

**DOI:** 10.3791/1137

**Published:** 2009-05-01

**Authors:** Christina A. Pacak, Douglas B. Cowan

**Affiliations:** Department of Anesthesiology, Children’s Hospital Boston and Harvard Medical School; Perioperative and Pain Medicine, Children’s Hospital Boston and Harvard Medical School

## Abstract

Despite the fact that electronic pacemakers are life-saving medical devices, their long-term performance in pediatric patients can be problematic owing to the restrictions imposed by a child's small size and their inevitable growth. Consequently, there is a genuine need for innovative therapies designed specifically for pediatric patients with cardiac rhythm disorders. We propose that a conductive biological alternative consisting of a collagen-based matrix containing autologously-derived cells could better adapt to growth, reduce the need for recurrent surgeries, and greatly improve the quality of life for these patients. In the present study, we describe a procedure for incorporating primary skeletal myoblast cell cultures within a hydrogel matrix to fashion a surgically-implantable tissue construct that will serve as an electrical conduit between the upper and lower chambers of the heart. Ultimately, we anticipate using this type of engineered tissue to restore atrioventricular electrical conduction in children with complete heart block. In view of that, we isolate myoblasts from the skeletal muscles of neonatal Lewis rats and plate them onto laminin-coated tissue culture dishes using a modified version of established protocols^[2, 3]^. After one to two days, cultured cells are collected and mixed with antibiotics, type 1 collagen, Matrigel™, and NaHCO_3_. The result is a viscous, uniform solution that can be cast into a mold of nearly any shape and size^[1, 4, 5]^. For our tissue constructs, we employ type 1 collagen isolated from fetal lamb skin using standard procedures^[6]^. Once the tissue has solidified at 37°C, culture media is carefully added to the plate until the construct is submerged. The engineered tissue is then allowed to further condense through dehydration for 2 more days, at which point it is ready for *in vitro* assessment or surgical-implantation.

**Figure:**
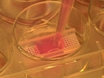


## Protocol

### Part 1: Assemble construct casting molds

Use a razor blade to halve silicone tubing (VWR) and cut it into 3 cm long pieces.
Place a drop of implant-grade RTV silicone adhesive (Rhodia) on the inside of each end of the tubing.
Quickly place a small piece (1 cm x 1 cm) of polyester mesh (McMaster-Carr) on the silicone adhesive drop and align it with the end of the tubing. This will provide a slightly raised and flat surface for construct attachment. Repeat for the other end.
Allow the mold to dry at room temperature for 3 days. It is helpful to make 20 to 30 molds at a time, as these are essentially disposable.
Once the adhesive is completely dry, store these molds in a beaker filled with 70% ethanol until they are needed. This step will help sterilize the molds and the finished product is shown in Figure 1.

### Part 2: Preparation for myoblast cell isolation

Dilute 1 mg Laminin (Sigma) in 250 mL 0.22 μm filter-sterilized phosphate-buffered saline (PBS) containing 1% Penicillin/Streptomycin (Invitrogen) and 1% Fungizone (Invitrogen).
Coat 150 mm tissue culture plates (BD Falcon) with 4 mL of the diluted Laminin solution from step 1.1 and incubate at 37ºC for at least 4 hours before plating the primary skeletal myoblasts.
Make the myoblast medium by mixing Ham’s F-10 Nutrient Mixture (Sigma) with 20% FBS (Atlanta Biologicals), 5 ng/L basic Fibroblast Growth Factor (Promega), 1% Penicillin/Streptomycin (Invitrogen), and 1% Fungizone (Invitrogen).
Prepare the skeletal muscle digestion solution by dissolving 1 g of ~295 U/mg Collagenase 2 (Worthington) in 100 mL 2.4 U/mL Dispase-2 (Roche). Add 0.037 g CaCl_2_ and mix well. Filter sterilize the digestion buffer by using a 0.2 µm filter disk fitted onto a 10 mL syringe and store aliquots at -20°C. Place the amount needed for tissue digestion at 37°C along with the myoblast media.

### Part 3: Myoblast isolation from skeletal muscle

Using forceps and small scissors, remove paraspinal muscles from dead neonatal Lewis rats by slicing down the back along the spinal cord, peeling back the skin and fascia layers and then excising the muscles.
In a tissue culture hood, place the excised muscles in a 50 mL conical tube (BD Falcon) filled with 40 mL of 1X Hanks Balanced Salt Solution (HBSS) (Invitrogen) on ice. Allow the muscle strips to settle to the bottom of the tube and carefully remove most of the liquid and blood remnants by vacuum suction with a sterile Pasteur pipette in a culture hood (Baker).
Pour the harvested muscle pieces onto a 150 mm culture dish and remove as much of the remaining liquid as possible with suction. Using 2 single-edged razor blades mince the tissue to a thick paste. Pour pre-warmed digestion solution on the paste and transfer the mixture to a fresh 50 mL tube using a 10 mL pipette. During the enzymatic digestion, the tissue is occasionally triturated (without bubbling) with a 10 mL pipette fitted onto a cordless Pipette-Aid (Drummond). Again, allow the tissue to settle and remove the excess liquid.
Place the tube on a rocking platform and digest at 37ºC for about 30 min.
Once the muscle is liquefied, pass the solution through a 70 μm cell strainer (BD Falcon) and centrifuge at room temperature for 10 min at 600 x g (Beckman GP). With a Pasteur pipette and suction, remove as much of the liquid as possible and resuspend the pellet with 10 mL of warm myoblast medium.
Pre-plate the cells on an uncoated 150 mm tissue culture plate for 15 minutes to help remove fibroblasts.
Transfer the cells that have not yet attached to the laminin-coated cultureware and dilute so that there is one plate for every 1 to 2 rat pups. Place the plate in a humidified incubator set at 37 °C with 5% CO_2_.

### Part 4: Casting engineered tissue constructs (5 mL)

After 1 to 2 days, remove 10 of the 150 mm myoblast tissue culture plates from the incubator, rinse them twice with 37°C 1X PBS, and add 4 mL of warm 0.05% Trypsin-EDTA to each plate. Return the plates to the incubator for 5 min.
Harvest the detached myoblasts from the plates in the culture hood and collect the liquid in a 50 mL tube (BD Falcon). Use 10 mL of myoblast medium to rinse all of the plates and combine this with the cells in the centrifuge tube. Pellet the cells at room temperature for 10 min at 600 x g (Beckman GP) and remove excess liquid as described in step 3.5.
Resuspend the myoblast cells in 1.6 mL of myoblast medium and place on ice.
Using forceps, carefully remove 4 construct molds from step 1.5 and rinse them twice with 1X PBS.  Vacuum suction off all traces of PBS with a Pasteur pipette before placing each mold in a 6-well tissue culture plate (BD Falcon).
Gather the other reagents needed for construct preparation (10X Ham’s F10, antibiotics, type 1 collagen [3.9 mg/mL]^[6]^ and Matrigel™) on ice and keep myoblast media at 37ºC. Combine 500 μL 10X Ham’s F10, 100 µL Penicillin/Streptomycin, 100 µL Fungizone, 1.6 mL collagen, and 750 µL Matrigel™ in a 15 mL conical tube. Mix the solution for 10 sec using a Mini-Vortexer (VWR) set to 10 and place on ice.
Next, add 80 µL of NaHCO_3_ and the myoblast suspension from step 3.3 to the mixture from step 3.5. Vortex the entire mixture for 10 sec and return the tube to the ice bucket for a few min to clear the solution of bubbles.
Carefully cast the mixture into the molds before it begins to solidify using a 10 mL pipette. Each mold will hold approximately 1 mL of the viscous liquid. Try to avoid introducing air bubbles during this procedure.
Carefully transfer the plate containing the cast constructs to the incubator. After ~30 min, remove the solidified constructs from the incubator and carefully add myoblast medium to each well to cover the tissue construct. Return the plate to the incubator (Figure 2).

### Part 5: Representative results

When this protocol is properly executed, the myoblast-containing tissue construct is ready for *in vivo* implantation (i.e. when removed from the mold) or for further *in vitro* analyses after 2 days.


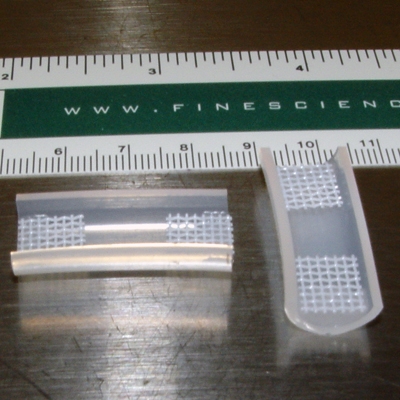


**Figure 1. **Examples of completed construct molds (see Step 1.5).


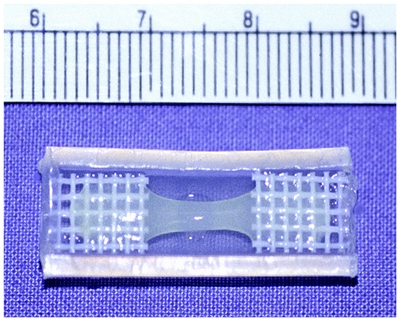


**Figure 2. **A solidified myoblast-containing tissue construct^[1]^.


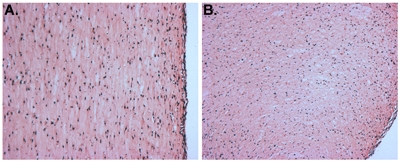


**Figure 3. **H&E stained sections of a myoblast-containing tissue construct. "A" depicts a longitudinal section and "B" shows a cross-section.

## Discussion

The molds in which the tissue construct will be cast can be made in any shape and size; however, there needs to be at least two points of attachment. Otherwise, the matrix and cells form a spherical structure and the cells die. In the present protocol, we describe the use of a polyester mesh for this purpose, yet we have also successfully used stainless steel mesh. Obviously, larger molds will require more cells and a larger volume of the other ingredients. When making the molds, it is important to minimize the amount of silicone adhesive used and to ensure that it is located at the very ends of the tubing. This is because myoblasts near the adhesive tend not to be viable, even after several days of curing. In addition, it is prudent to plate the myoblasts for only a day or two before preparing the tissue constructs as contaminating fibroblasts will multiply rapidly and eventually overwhelm the myogenic components of the culture. Similarly, myoblasts should not be plated densely because cells in contact with one another will begin to fuse and differentiate into myotubes. In regard to the fabrication of the tissue constructs, there are a number of commercially available sources of type 1 collagen; however, each demonstrate differences in the rate of solidification and the consistency of the final product. Furthermore, it is essential that the collagen preparation is not irradiated with UV light as this process inhibits the gelation process. In our hands, the constructs change color from a dark pink to a light pink during solidification. Finally, our collagen preparation is acid-solublized (not pepsin digested), so the pH of the mixture in step 3.5 needs to be increased by adding NaHCO_3_ before incorporating the cells.
